# Encephalo-Spinal Meningitis

**Published:** 1843-04

**Authors:** 


*Encephalo-Spinal Meningitis.*—Dr. Bell, editor of the *Bulletin of Medical Science*, designates by this term, the disease described by Dr. Richardson, of Tenn., in the last vol. of this Journal (p. 430, Dec. 1842); and, after quoting Dr. R's account, gives the following notice of a discussion in the French Royal Academy of Medicine (sitting of the 6th September last) on a similar disease that prevailed in different parts of France.

"The subject of the disease described by Dr. Richardson, was brought up in a meeting of the French Royal Academy of Medicine by a Report from M. Ferrus, on a memoir sent by M. Rollet, chief physician to the military hospital at Nancy. It is well known that the epidemic meningitis prevailed in different parts of France, viz: at Versailles, Bordeaux, Avignon, Strasburg, &c. Interesting accounts of the disease have already appeared, by Faure, Villars, Chauffard, Forget, &c. It showed itself in Nancy with most severity among the military. Rollet regards it under two aspects, and as constituting two varieties: *cerebro-spinal meningitis* and *encephalo-meningitis*, according as the envelopes alone of the nervous centres, or the cerebral substance itself participate in the inflammation. At Nancy, as elsewhere, the disease manifested itself by symptoms of great violence; there having been present the same disorder, in the meninges, active congestion, suppuration, and even softening of the brain and spinal marrow, according to the degree and intensity of the disease, as exhibited in *post-mortem* examinations of those who sank under it. The symptoms were, also, very much alike in different places in which the epidemic made its attacks, modified, however, by the seat of the inflammation;—a point on which Rollet lays considerable stress.

The subject of treatment is of course important, and it has been carried out according to the view of the pathology of the disease entertained by different practitioners. At Bordeaux, the disease having manifested some symptoms of periodicity, sulphate of quinine combined with opium was much thought of. At Avignon, Chauffard derived from opium, results described as truly miraculous. After some tentative practice, Rollet hit upon a plan which was successful, in his hands, but which he had recourse to only in cases of encephalo-meningitis. It is cauterization of the spine with red-hot iron. He used the cautery in such a manner as to give rise to a burn of the second degree, along the depression on each side of spinous process; repeating several times this energetic practice. In most cases the sensibility which had been deadened was soon revived; the pulse recovered its force and fullness, and, in fine, reaction followed sufficient to allow of bleeding the patient,—with a frequency adapted to the indications of the case. In two patients, symptoms of intermission having appeared, sulphate of quinine was employed. One of these persons died; the other recovered.

The following is a summary of the therapeutical course pursued by Rollet:—Fourteen cases of simple cerebro-spinal meningitis were treated with antiphlogistics alone; and of these all recovered. Of the same number of persons (14) attacked with encephalo-meningitis, four were subjected to the ordinary treatment, all of whom perished. The other ten were treated by cauterisation, with a result of six cures and four deaths.

The reporter to the Academy, Ferrus, eulogised the manner in which the subject had been treated by Rollet, and proposed in consequence a letter of thanks, the remission of his memoir to the committee of publication, and the inscription of his name on the list of candidates for corresponding membership.

An animated discussion in the Academy followed the report, in which Rochoux, Castel, Honoré, &c., took a part.

These gentlemen thought that Rollet had committed an error in diagnosis. The extreme gravity and indeed almost constant mortality from meningitis were well known; and how then explain this with the success in treatment as related? Probably, continue the objectors, the disease was a pernicious (congestive) fever, the paroxysms of which run into each other so as to simulate a continued fever. Some cases of rheumatism were also confounded with the reigning malady. Bousquet, in proof of the epidemic not being an open, ordinary phlegmasia, referred to the success which Chauffard had with the employment of opium. In fact, this physician, alarmed at the results of a simple antiphlogistic treatment, went over, once more, a study of all the phenomena of the disease, and detected a predominance of disorder of the nervous system which called for the use of narcotics.

To all these critiques Ferrus replies, by asserting that there was no mistake in the diagnosis. Rollet, as well as Forget, and a number of other distinguished observers in the departments, did indeed at first suppose the existence of a pernicious fever; but all of them, after an attentive and conscientious investigation, were led to form an opposite opinion. Now, when a number of enlightened men agree, without prior concert, in the same opinion, there are strong grounds for our admitting that their view is the true one. The number of cures is objected to in connexion with the nature of the disease described; but the numbers furnished by Rollet are well authenticated, and are proved by proper documents duly detailed. But, besides,—ought we to compare that which passes in one locality, or during an epidemic, with that which transpires in another locality, and when the disease is sporadic? And, again, Baudelocque remarked, that what was said of the almost uniform mortality of meningitis, applies to the granular kind in children and not to the simple meningitis of adults. The conclusions of the report were adopted by the Academy.

